# Post-COVID-19 Femoral Head Osteonecrosis Exhibits Mast Cell Clusters, Fibrosis, and Vascular Thrombosis: Key Pathological Mechanisms in Long COVID-19 Bone Degeneration

**DOI:** 10.3390/pathophysiology32030036

**Published:** 2025-07-18

**Authors:** Asya Kuliyeva, Natalia Serejnikova, Gulnara Eshmotova, Yulya Teslya, Anastasia Ivina, Alexey Zarov, Michael Panin, Alexey Prizov, Vera Lyalina, Dmitry Shestakov, Alexey Fayzullin, Peter Timashev, Alexey Volkov

**Affiliations:** 1 Department of Pathological Anatomy, Medical Institute, Patrice Lumumba Peoples’ Friendship University of Russia, 117198 Moscow, Russia; aerzieva@mail.ru (A.K.); geshmotova@mail.ru (G.E.); julia.teslyaaa@yandex.ru (Y.T.); ivina_aa@pfur.ru (A.I.); i@bonelab.ru (A.V.); 2 Institute for Regenerative Medicine, Sechenov First Moscow State Medical University, 119991 Moscow, Russia; fayzullin_a_l@staff.sechenov.ru (A.F.); timashev_p_s@staff.sechenov.ru (P.T.); 3 Department of Traumatology, Orthopedics, and Disaster Surgery, N.V. Sklifosovsky Institute of Clinical Medicine, Sechenov First Moscow State Medical University, 119435 Moscow, Russia; zarov_a_yu@staff.sechenov.ru; 4 Department of Traumatology and Orthopedics, Medical Institute, Patrice Lumumba Peoples’ Friendship University of Russia, 117198 Moscow, Russia; panin-mihail@yandex.ru (M.P.); aprizov@yandex.ru (A.P.); 5 Department of Hospital Therapy Named After Academician G.I. Storozhakov Medical Faculty, Pirogov Russian National Research Medical University, 117513 Moscow, Russia; vera_lyalina@mail.ru; 6 Loginov Moscow Clinical Scientific Center, 111123 Moscow, Russia; dimitrauma@bk.ru

**Keywords:** osteonecrosis, femoral head, long COVID-19, articular tissues, pathomorphology, mast cells, fibrosis, thrombosis, giant cells

## Abstract

**Background/Objectives:** Osteonecrosis of the femoral head (ONFH) is a common condition in hip surgery, which is characterized by the death of bone cells due to disruption of the blood supply and ultimately irreversible destruction of the hip joint. As a result of the COVID-19 pandemic, a significant increase in the incidence of ONFH has been identified. To better understand the pathogenesis of ONFH in the context of COVID-19, our research aimed to determine pathomorphological changes in articular tissues specific to post-COVID-19 ONFH. **Methods:** Using morphological, morphometric, and statistical methods, the femoral heads after hip arthroplasty were retrospectively studied in patients with post-COVID-19 ONFH (*n* = 41) compared to a non-COVID-19 group of patients (*n* = 47). **Results:** Our results revealed that the key morphofunctional biomarkers of post-COVID-19 ONFH were clusters of mast cells, extensive areas of fibrosis, numerous arterial and venous thrombi, and giant cell granulomas. The potential relationship of those morphological features with the action of the SARS-CoV-2 coronavirus was discussed. **Conclusions:** Mast cells have been proposed as the leading players that may trigger the main molecular and cellular mechanisms in the development of post-COVID-19 ONFH and can be considered a diagnostic sign of the disease.

## 1. Introduction

Osteonecrosis, also known as aseptic (avascular) necrosis of bone tissue, is a severe chronic disease with multifactorial etiology affecting young and middle-aged people (30–50 years) and associated with the death of bone cells in the case of circulatory disorders [1]. Osteonecrosis most often develops in the femoral head and is characterized by rapid progression with increased chronic pain, leading in 2–4 years to a significant limitation of motor activity, to a collapse of the subchondral bone and the development of secondary arthritis of the hip joint with subsequent disability in patients [2,3]. Osteonecrosis of the femoral head (ONFH) causes deep suffering in patients and leads to significant social and economic problems worldwide. According to clinical and epidemiological studies in Russia, the incidence of ONFH is 166 cases per 100,000 population [4]; in Europe and the United States, this disease was detected in about 600,000 people; and in China, it was detected in about 8 million people [5]. To date, there is no consensus on effective strategies for treating ONFH: both conservative and surgical methods have a number of limitations [6,7]. The prognosis for patients is largely determined by early detection and timely treatment before the onset of the collapse of the femoral head; therefore, the analysis of the mechanisms of the pathogenesis of ONFH is the main issue currently considered by Russian and foreign scientists [5,8].

Multiple risk factors, toxins, and events can trigger ONFH, including fractures, steroid therapy, alcohol abuse, inflammatory diseases, and genetic factors [9]. It should be noted that the mechanisms of ONFH with different etiologies vary greatly, although patients sometimes have similar clinical manifestations and prognoses. Impaired necrotic trabecular bone integrity in steroid-induced ONFH has been shown to be associated with increased bone resorption and in alcohol-induced ONFH to be associated with reduced osteogenesis [10]. The pathophysiological mechanisms underlying ONFH are not completely clear, and they are complex and diverse, including impaired blood flow with angiogenesis disorder, coagulopathy and endothelial dysfunction, lipid metabolism disorder, intraosseous hypertension, and osteoporosis [11,12,13,14]. As a result, histopathological and microarchitectural disorders develop, which are hypomineralized bone tissue, its remodeling and destruction with replacement by fibrous tissue, necrosis of osteocytes, bone marrow necrosis and its fat transformation, thinning and degeneration of the cartilage zone, hyperplasia of synovial tissue, and chronic inflammation [2,15,16,17].

SARS-CoV-2 coronavirus infection, the culprit of the COVID-19 pandemic, is associated with significant long-term consequences (post-COVID-19 syndrome) for various organ systems, including the musculoskeletal system in the form of joint pain and muscle weakness [18,19]. Additionally, the combination of immune-mediated vascular damage in COVID-19 and the routine use of systemic corticosteroid therapy has recently led to a sharp surge in ONFH [20,21]. Given the scale of the spread of COVID-19 infection around the world, identifying and treating this form of ONFH is of critical social and economic importance. In this regard, the literature is now actively discussing the potential relationship between coronavirus infection, corticosteroid therapy, and the development of ONFH [22,23,24]. Scientific data are widely presented, focusing mainly on the features of clinical manifestations of post-COVID-19 ONFH [25,26,27,28]. However, the specific histopathological features of this form of ONFH are not yet clear. Increasing awareness of the morphological features of post-COVID-19 osteonecrosis will increase understanding of the mechanisms of pathogenesis of this disease, which in turn may be crucial in making treatment decisions and mitigating potential complications. In this regard, the purpose of our work was to identify the structural and functional features of the articular tissues of the femoral heads during osteonecrosis in patients who have undergone coronavirus infection, compared with this pathology in patients without COVID-19 and to determine the potential relationship of these morphological signs with the action of the SARS-CoV-2 coronavirus.

## 2. Materials and Methods

### 2.1. Study Object

Preparations of femoral heads obtained after total arthroplasty in 88 patients with diagnosed ONFH at an advanced stage for the period 2017–2023 were retrospectively examined. The inclusion criteria for this study were as follows: patients between 22 and 70 years with severe hip pain and radiological signs of ONFH of various etiologies (trauma-induced, steroid-induced, alcohol-induced, idiopathic), stage 3–4 according to ARCO (Association Research Circulation Osseous). All patients were divided into 2 groups: a control non-COVID-19 group (*n* = 47), consisting of patients with ONFH diagnosed in 2017–2018 before the coronavirus appeared, and an experimental post-COVID-19 ONFH group (*n* = 41), with the rRT–PCR test confirming coronavirus infection. The incidence of comorbidities as additional risk factors of ONFH was minimal in the post-COVID-19 ONFH group. The treatment of patients in both groups did not differ and was carried out in accordance with the National Clinical Guidelines for the treatment of ONFH, without adjusting for the effect of coronavirus infection on the development of ONFH.

### 2.2. Morphological Analysis

Femoral head samples were fixed in 10% neutral buffered formalin (Biovitrum, Moscow, Russia). Each head was then sawn into thin longitudinal sections 5 mm thick, and at least 3 sections from each head with significant necrotic, sclerotic, and fibrous changes were selected for further analysis. The samples were then decalcified in SoftiDek solution (Biovitrum, Moscow, Russia), dehydrated in isopropyl alcohol in an Epredia STP120 spin tissue processor (Thermo Fisher Scientific, Waltham, MA, USA) and embedded in paraffin using a HistoStar embedding workstation (Thermo Fisher Scientific, USA). Sections that were 4 μm thick were made on a Leica RM 2125RTS microtome (Leica Microsystems, Wetzlar, Germany), stained with hematoxylin and eosin (Biovitrum, Russia), Mallory (Biovitrum, Russia), safranin O/methyl green (Biovitrum, Russia) for the detection of glycosaminoglycans in cartilage matrix and in mast cells granules according to standard protocols. Histopreparations were digitized on a NanoZoomer S20MD histoscanner (Hamamatsu, Saitama, Japan) for morphological analysis.

### 2.3. Morphometric Analysis

In the NDP.view2 program (Hamamatsu, Japan), whole slide images per 1 mm^2^ of section area were evaluated: thickness of articular cartilage, areas of necrotic altered bone trabeculae and unchanged bone trabeculae, area of fibrous tissue, area of cartilage and bone regenerates, number of thrombosed blood vessels, mast cells, and giant multinucleated cells. Morphological evidence of cartilage destruction and dystrophy, as well as a decrease in the content of glycosaminoglycans in the cartilage matrix in each sample was assessed by Mankin’s method in 5 different visual fields at 200-fold magnification [29].

### 2.4. Statistical Analysis

The statistical analysis of experimental data was performed using GraphPadPrism 10.00 software for Windows (GraphPad Software, San Diego, CA, USA). Distribution normality was determined by the Shapiro–Wilk test. The results of semi-quantitative histological scoring were analyzed by using the Kruskal–Wallis test followed by Dunn’s multiple comparison test. Intergroup differences in quantitative data were analyzed by the one-way ANOVA method followed by Tukey’s multiple comparison test. The results of the statistical analysis were presented as bar charts of means and standard deviations for quantitative data and as median values and interquartile range for scoring parameters. *p*-values ≤ 0.05 were considered statistically significant.

## 3. Results

An extensive analysis of clinical and demographic features among patients diagnosed with ONFH across the studied groups was conducted and is presented in Table 1. In the non-COVID-19 group, the mean age of the patients was 53.7 ± 9.3 years, with a male-to-female ratio of 1.7:1, and the mean BMI (body mass index) was 29.7 ± 5.2 kg/m^2^. The mean duration of ONFH symptoms was 25.4 months. Among the studied etiologies of osteonecrosis, the traumatic-induced form prevailed (45.6%). Concomitant diseases were observed in only 12.8% of the participants. The distribution in the post-COVID-19 group showed a 1.4: 1 ratio, the mean age of participants was 51.2 years ± 10.6 years, and the mean BMI was 28.6 ± 2.7 kg/m^2^. The mean interval from COVID-19 to the onset of ONFH was 11.3 months. Among the patients, 83.2% exhibited mild to moderate COVID-19 symptoms and received care either at home or in the hospital without steroid administration, while 16.8% had severe COVID-19 requiring steroid therapy during inpatient treatment. A significant proportion of the patients (92.7%) showed no endocrine diseases, blood disorders, musculoskeletal abnormalities, or malignant factors contributing to ONFH. Comparative intergroup analysis showed no statistically significant differences in the majority of parameters between patients with post-COVID-19 ONFH and those with other forms of non-COVID-19 ONFH. However, patients in the post-COVID-19 differed statistically significantly by the duration of ONFH before total hip arthroplasty in contrast to the non-COVID-19 group (2 times shorter, *p* = 0.03).

**Figure 1:**
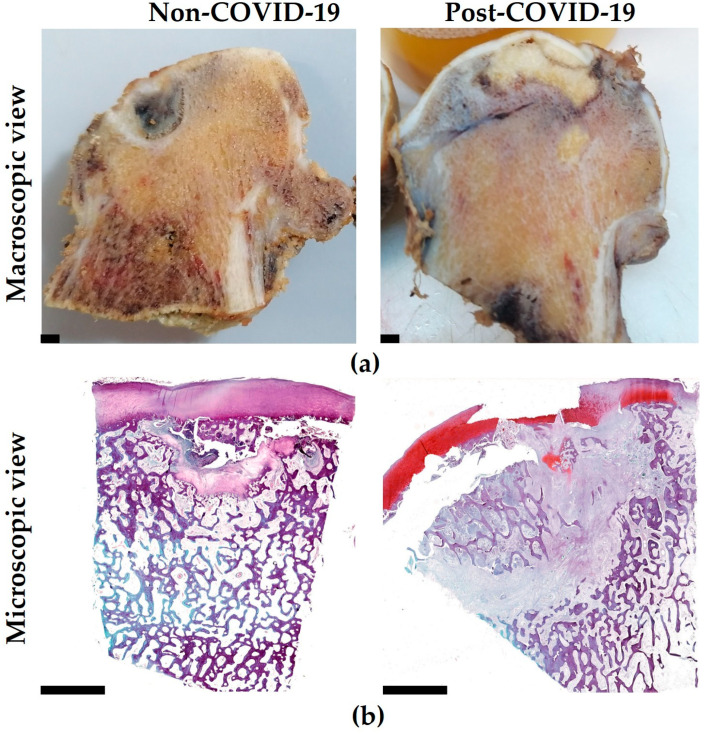
Gross and histological examination of post-COVID-19 femoral heads with osteonecrosis in comparison with the non-COVID-19 samples. (**a**) Femoral head contour deformation, subchondral bone necrosis and articular cartilage defects observed in both groups; (**b**) more extensive necrotic areas and more significant fibrosis detected in the post-COVID-19 group. Scale bar—5 mm.

Macroscopic evaluation of non-COVID-19 and post-COVID-19 samples revealed in both groups a typical appearance of ONFH with head contour deformation (flattening and thinning), with clearly delimited areas of subchondral bone necrosis and impaired structural integrity of articular cartilage above the necrotic zone (Figure 1a). Moreover, in the case of post-COVID-19 ONFH samples, compared with the non-COVID-19 group, more extensive necrotic areas and more significant fibrosis were observed, replacing the affected areas of bone (Figure 1b).

**Figure 2:**
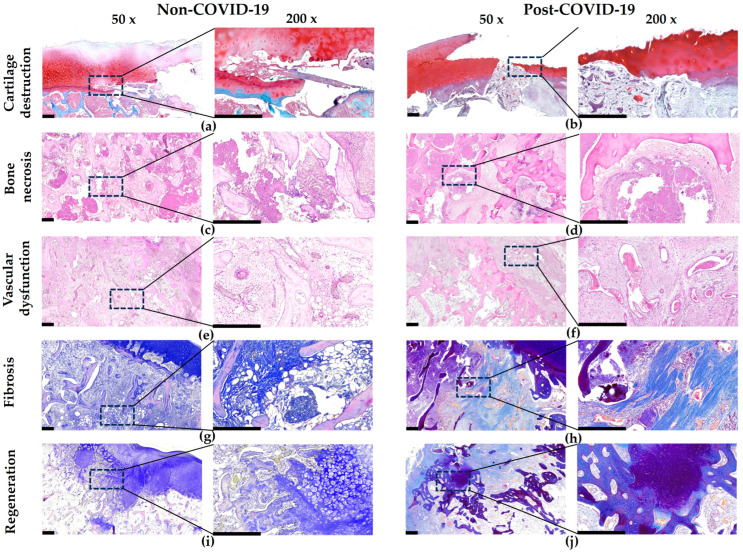
Histological analysis of pathomorphological changes in articular tissues of post-COVID-19 ONFH samples compared to the non-COVID-19 group. (**a**,**b**) Similar articular cartilage lesions in both groups, safranin O and methyl green staining. (**c**,**d**) Similar necrotic-affected areas in both groups, hematoxylin and eosin staining. (**e**) Arterial thrombosis in the non-COVID-19 ONFH group, hematoxylin and eosin staining. (**f**) Arterial and venous thrombosis in the post-COVID-19 ONFH group, hematoxylin and eosin staining. (**g**) Fibrosis with less tightly and multidirectional arrangement of collagen fiber bundles in the non-COVID-19 ONFH group, Mallory staining. (**h**) Fibrosis with formation of longitudinally oriented strands of dense fibrous tissue in the post-COVID-19 ONFH group, Mallory staining. (**i**,**j**) Similar reparative regeneration areas in both groups, Mallory staining. Scale bar—500 μm.

**Figure 3:**
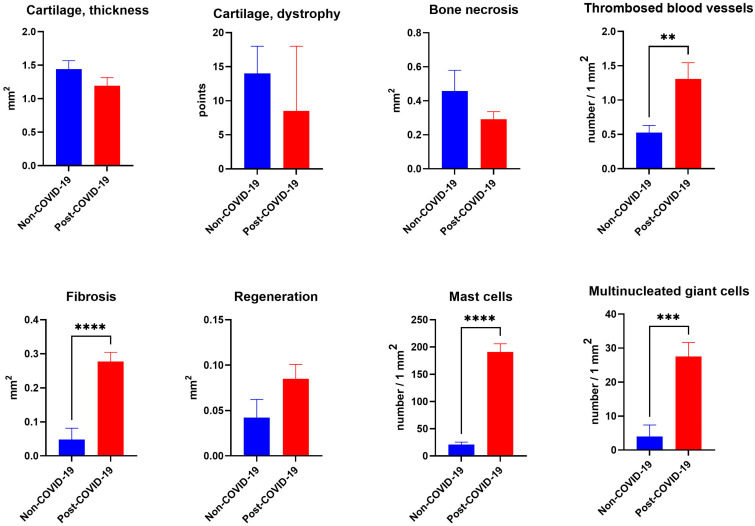
Morphometric analysis of pathomorphological changes in articular tissues of post-COVID-19 ONFH samples compared to the non-COVID-19 group; **—*p* < 0.01; ***—*p* < 0.001; ****—*p* < 0.0001.

According to microscopic analysis, the articular cartilage of the femoral heads of patients of both groups was characterized by destruction, focal areas of thinning, or its complete absence above the necrotic zone (Figure 2a,b 50×). In relatively preserved cartilage, signs of the osteoarthritic stage of cartilage degradation were observed: surface villosity with the presence of cracks of different depths, dystrophic changes in chondrocytes with the formation of numerous clones, zonal hypo- and hypercellularity, and a decrease in the content of glycosaminoglycans in the cartilage matrix. In the deep cartilage zone, the frequent formation of small cysts (tunnels) was noted, the tidemark line became intermittent, and the layer of calcified cartilage was sharply thinned (Figure 2a,b 200×). In a semi-quantitative score of these parameters in articular cartilage, statistically significant differences were not found between groups, and there was also no difference in cartilage thickness (Figure 3).

The necrotic sites in both groups were of different sizes and affected both trabecular bone and bone marrow, forming cell-free necrotic mass-filled sites and residual bone trabeculae with empty lacunae (Figure 2c,d). The bone trabeculae around necrotic masses were atrophied and fragmented, with numerous microcracks (Figure 2c 200×). The necrotic masses were isolated by newly formed fibrous tissue like a type of sclerotic ring separating the necrotic zones from the rest of the head tissues (Figure 2d 200×). No statistically significant results on the size of necrotic zones could be identified (Figure 3).

In the necrotic-affected areas, reparative chondro- and osteogenesis was observed in both groups (Figure 2i,j). At the same time, there were no statistically significant differences in the area of bone and cartilage reparative regeneration between the groups (Figure 3).

**Figure 4:**
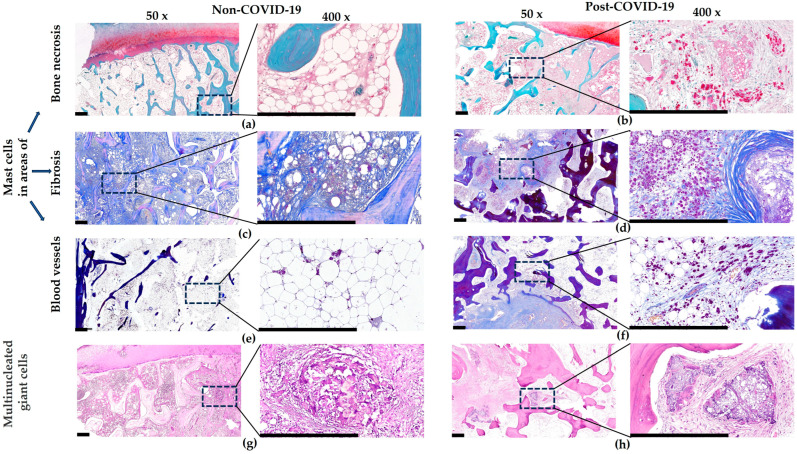
Microscopic assessment of clusters of mast cells and giant cells prevalent in post-COVID-19 ONFH samples compared to the non-COVID-19 group. (**a**,**b**) Mast cell clusters around foci of necrotic bone trabeculae, safranin O/methyl green staining. (**c**,**d**) Mast cell clusters in fibrous tissue, Mallory staining. (**e**,**f**) Mast cell clusters around blood vessels, Mallory staining. (**g**,**h**) Giant cell clusters around foci of necrotic bone trabeculae, hematoxylin and eosin staining. Scale bar—500 μm.

Common dystrophic changes in blood vessels to both groups were thrombosis, perivascular fibrosis, and vasculitis (Figure 2e,f). At the same time, the distinguishing features of post-COVID-19 ONFH samples compared with the non-COVID-19 ones were a higher level of thrombosis (2.6 times, Figure 3) involving not only arterial, but also venous blood vessels (Figure 2f), as well as numerous perivascular clusters of mast cells (Figure 4).

Extensive areas of trabecular and bone marrow necrosis were gradually replaced by fibrous tissue. In the post-COVID-19 ONFH group, the relative area occupied by fibrous tissue was 5.6 times higher than this parameter in the non-COVID-19 group (Figure 3). It is also noteworthy that fibrillogenesis differed in post-COVID-19 samples and was characterized by the formation of longitudinally oriented strands of dense fibrous tissue (Figure 2h), and sometimes nodules, in their architectonics resembling connective tissue in hypertrophic skin scars and was characterized by significant compactization of collagen fibers with some moire patterns. However, in the non-COVID-19 samples, the bundles of collagen fibers in fibrous tissue were located less tightly and were multidirectional (Figure 2g).

The main specific feature of the post-COVID-19 femoral heads we identified compared to the non-COVID-19 group was a sharp increase (9.1 times) in the content of mast cells (Figure 3). Thus, particularly large accumulations of these cells were observed around blood vessels, in foci of fibrosis, and also near areas of necrosis of bone trabeculae and bone marrow (Figure 4b,d,f). In the non-COVID-19 samples, on the contrary, only single mast cells were found and significant focal lymphocytic infiltration attracted attention (Figure 4a,c,e).

Additionally, an interesting feature of osteonecrotic heads of both groups was focal clusters of giant multinucleated cells phagocytizing necrotic fragments (Figure 4g,h). Morphometric analysis showed that the content of giant cell granulomas in the post-COVID-19 group was significantly increased (6.9 times) compared with the non-COVID-19 group (Figure 3).

## 4. Discussion

In our work, the morphological picture of the post-COVID-19 form of ONFH was studied and its specific features were identified, with a high probability due to the influence of COVID-19 infection. These morphological features consisted in a sharply increased content of mast cells, much larger areas of bone marrow fibrosis, an increase in the level of thrombus formation, and an increase in the number of giant cell granulomas. The specific morphological changes we identified in the post-COVID-19 articular tissue samples were absent or significantly lower compared with non-COVID-19 histological samples with diagnosed ONFH of various etiologies, including steroid-induced necrosis. In addition, in the post-COVID-19 group, most of the patients (83.2%) had milder to moderate forms of COVID-19 and underwent outpatient treatment without steroids, and the specific morphological features in their samples did not significantly differ from the samples of patients with steroid therapy. When comparing the study groups statistically, there were no notable differences in most demographic and clinical parameters between COVID-19 patients and those with different etiologies of non-COVID-19 ONFH. Additionally, in the post-COVID-19 group, most of the patients (92.7%) had no history of any endocrinological, hematological, musculoskeletal, or malignant causes of ONFH. The observed evidence allows us to propose that the morphological changes detected in the post-COVID-19 group are likely attributed to the COVID-19 virus itself and not to steroids used to treat COVID-19 or other risk factors for ONFH.

Of particular interest is the previously undescribed influence of mast cells in the development of post-COVID-19 osteonecrosis. We detected significant accumulations of these cells mainly near blood vessels, in foci of fibrosis, and also near necrotized areas of bone trabeculae and bone marrow. While commonly linked to allergic reactions, mast cells demonstrate their importance as vital sensory and effector components of the immune system, influencing a broad spectrum of physiological and pathological states [30,31]. Current research highlights the substantial influence of these cells on bone metabolic processes and their involvement in the etiology of multiple musculoskeletal diseases, such as different types of arthritis, osteoporosis, and tendinopathy [32]. Found in the bone marrow, mast cells act as producers of extensive mediator profiles, releasing them promptly when mature cells are stimulated following their differentiation processes in mucosal or connective tissues. Among these mediators, several exhibit osteocatabolic capabilities by enhancing osteoclast production (histamine, IL-6) and/or suppressing osteoblast processes (IL-1, TNF). Tryptase released by mast cells also induces the activation of MMPs acting to destruct vital extracellular matrix proteins like collagen and aggrecan. In contrast, mast cells can potentially act in an osteoprotective manner by stimulating osteoblasts (TGF-b) or reducing osteoclastogenesis (IL-12) [33]. In addition, by secreting particular cytokines such as TNF, IL-1, and IL-17, mast cells induce synovial fibroblast activation and regulate the apoptosis of these cells with the help of histamine and tryptase. The mutual interaction between these cell types is facilitated by SCF and IL-33, ultimately driving mast cell recruitment and subsequent activation [34]. It has been shown that there is a causal relationship between the induction of femoral head ischemia and the early onset hip synovitis through HIF-1 activation and increased IL-6 expression [35]. As is known, IL-6 is also secreted by mast cells and is one of the key pro-inflammatory cytokines that plays an important role in both initiating an acute inflammatory response and in maintaining a chronic response. Uncontrolled synovitis can lead to the gradual deterioration of articular structures, impacting cartilage and bone integrity, thereby aggravating the development of osteonecrosis.

The numerous arterial and venous thrombi that we identified in post-COVID-19 ONFH samples, in contrast to only arterial thrombi in ordinary ONFH, may also be due to the influence of significant perivascular accumulations of mast cells. Being located near blood vessels and secreting various vasoactive mediators (histamine, cytokines, proteases, chemokines, leukotrienes, etc.), activated mast cells cause microvasculature dysfunction and affect platelet adhesion and fibrin formation, leading to hypoxia, tissue edema, thrombosis and coagulopathy [36,37,38].

The fibrosis of post-COVID-19 ONFH samples, which we found to be significant in area and specific in collagenogenesis, is also most likely due to mast cell activation. The literature presents data on the formation of significant fibrous alterations in pulmonary tissue microenvironment of deceased COVID-19 patients, with foci of mast cell accumulation in fibrous tissue [39]. In this regard, the significance of mast cells in the processes of fibrillogenesis is currently being actively discussed. One pathway is classical mast cell degranulation, with the release of profibrotic mediators acting on fibroblast receptors and activating collagen synthesis. Another pathway is the recently discovered exosomal pathway, in which exosomes released by mast cells are absorbed by the fibroblast cytosol, which leads to proline hydroxylation and increased collagen production. Various components of the mast cell secretome trigger enhanced fibroblast proliferation, boost their migratory potential, facilitate their conversion into myofibroblasts characterized by alpha-smooth muscle actin accumulation, markedly stimulate collagen production, and promote epithelial–mesenchymal transition leading to further myofibroblast generation [40].

For example, through interaction with the PAR-2 receptor, mast cell tryptase stimulates both fibroblast proliferation and type I collagen synthesis [41], while chymase triggers the activation of matrix metalloproteinases (MMPs-9 and -2), thereby promoting extracellular matrix rearrangement [42]. Among mast cell mediators, TGF-β, IL-4, and IL-13 have been identified as key players in fibrosis pathogenesis [43]. Notably, recent studies have demonstrated an increase in the number of mast cells expressing carboxypeptidase A3 (CPA3) correlates with the development of fibrous tissue [39]. It is known that CPA3 plays a role in modulating endothelin-1 concentrations [44] while also participating in both the synthesis and breakdown of angiotensin II [45]—two important profibrotic factors; thus, mast cells can indirectly affect the development of fibrosis. Endothelin1 is a potent chemoattractant of fibroblasts, facilitating their differentiation into myofibroblasts and the formation of extracellular matrix components [46]. On the other hand, by activating fibroblasts, angiotensin II triggers their proliferation and subsequent extracellular matrix synthesis [47]; similarly, angiotensin II elicits apoptosis in alveolar epithelial cells, a crucial factor initiating the progression of pneumofibrosis [48]. Based on the accumulated data, the heightened inflammatory response in COVID-19 patients leads to enhanced mast cell activity and increased CPA3 expression, resulting in tissue damage and compromised repair processes that progress to fibrosis [49].

Every year, there is more and more evidence of the role of mast cells in the development of COVID-19 pathology. Post-mortem analysis of COVID-19 patient lung biopsies demonstrated extensive recruitment of mast cells to alveolar septa and pulmonary parenchyma in cases of coronavirus infection [50]. Additionally, the severity of COVID-19 and the degree of lung damage were positively correlated with increased alveolar mast cell presence, enhanced degranulation, and amplified inflammatory mediator profiles in patient serum compared to non-infected individuals [49]. In animal experiments, it was shown that mast cell degranulation induced by the coronavirus S protein led to the inflammation of bronchial, tracheal, and lung epithelial and endothelial cells [38], which triggered the upregulation of inflammatory mediators in endothelial cells of brain microvessels and disruption of their intercellular tight junctions, and also stimulated the inflammation of microglial cells, their activation, and their proliferation [51]. These results indicate that massive recruitment and triggering of mast cells is a feature of the pathogenesis of COVID-19 and can be considered as a pathological hallmark of this disease.

Currently emerging patterns demonstrate the extensive and varied roles of mast cells and their mediators in driving COVID-19 pathogenesis, modulating inflammatory processes, coagulopathy, and fibrosis. One of the likely mechanisms that is receiving much attention during SARS-CoV-2 development is the renin–angiotensin system. ACE2, or angiotensin-converting enzyme 2, degrades angiotensin II, a vasoactive substance with pro-inflammatory effects leading to acute pulmonary injury in individuals infected with SARS-CoV-2 [52]. Functioning as a viral receptor, ACE2 binds the SARS-CoV-2 S protein to facilitate cellular entry, while viral infection stimulates IFN production that enhances ACE2 activity [53]. The mechanism of viral penetration requires cellular proteases, with TMPRSS2 being a key player. It has recently been shown that IFN-α2 elevates surface levels of ACE2 and TMPRSS2 on human lung mast cells, concurrently triggering renin production in these cells. In addition, new evidence demonstrates the synergistic action of SARS-CoV-2 and IL-33 in stimulating human mast cells to produce and release proteolytic enzymes, such as chymase and tryptase, and pro-inflammatory cytokine IL-1, thereby promoting inflammation and cytokine storm development [54]. Through its interaction with the S protein, mast-cell-specific MCP2 chymase creates a complex that supports protease-dependent mechanisms of viral penetration [55]. Based on this, the authors proposed the following mechanism: infection with coronavirus causes an increase in IFN levels in the body, which, in turn, promotes the mast cell surface presentation of ACE2 and TMPRSS2, and mast cells become ready to be infected with the virus, which allows the virus to spread further, involving more and more of these cells and stimulating the powerful secretion of their effector molecules. In addition to ACE2, the SARS-CoV-2 coronavirus can use alternative cofactors to facilitate entry, such as histamine receptors. Apart from ACE2, the SARS-CoV-2 virus employs alternative entry facilitators, including histamine receptors. Findings reveal that HRH1 (histamine receptor H1) serves dual roles, functioning independently as a SARS-CoV-2 receptor and enhancing ACE2-dependent entry via direct engagement with ACE2 and the S-protein [56]. At the same time, it is known that numerous histamine receptors and, in particular, HRH1 are localized on the mast cell membranes, which contribute significantly to the development and regulation of allergic conditions triggered by histamine [57] and, according to the recent data, in the development of COVID-19 [37]. Thus, considering their role in cytokine-mediated receptor regulation in COVID-19 pathogenesis, mast cells may serve as a reservoir for SARS-CoV-2 infection [58].

COVID-19 infection may also be responsible for the increase in the content of giant multinucleated cells in osteonecrosis, as a number of authors have reported an increase in the frequency of giant cell arteritis in COVID-19 [59,60] and giant cell myocarditis [61,62]—the morphological feature of which is the presence of foci of giant multinucleated cells in the walls of the affected vessels and among the muscle fibers of the myocardium. The cellular mechanisms regulating the formation of such giant multinucleated cells are not yet clear. It is assumed that the CaMKII-like S protein system of SARS-CoV-2-infected cells promotes cell membrane fusion to further induce syncytial giant multinucleated cell formation [63]. As for the appearance of giant multinucleated cells in musculoskeletal diseases unrelated to COVID-19, there is evidence in the literature of abnormally large osteoclasts accompanied by the development of giant cell granulomas within bone marrow and synovium in rapidly developing osteonecrosis of the femur [64], as well as in osteonecrosis associated with Crohn’s disease [65]. It is suggested that such granulomas in osteonecrosis may reflect reparative processes in damaged bone tissue [66], which is consistent with our data on the best regenerative potential of COVID-19 samples. Additionally, numerous fibrous rings around necrotic areas can also, from a biomechanical point of view, be considered as an adaptive reaction in response to a decrease in elastic modulus in subchondral bone osteonecrosis [67]. Such formations may act as compensatory structural reinforcement in the femoral head [68].

## 5. Conclusions

**Figure 5:**
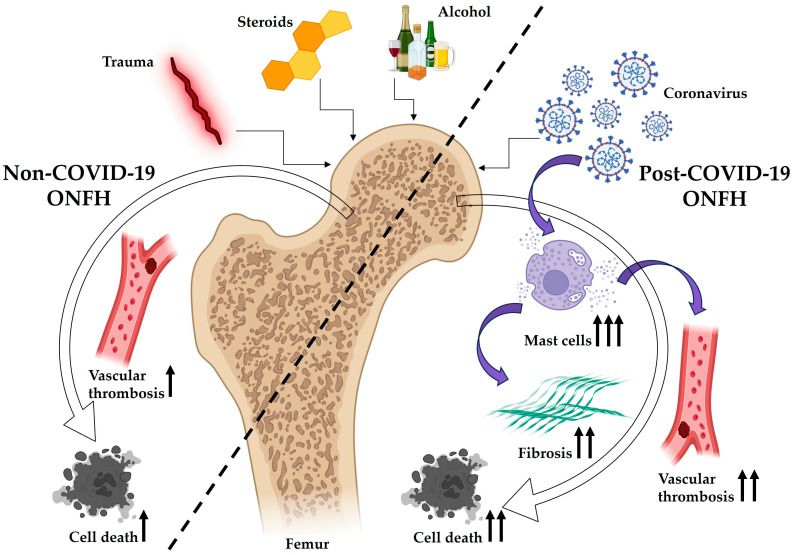
Schematic diagram showing the proposed role of mast cells in the pathogenesis of post-COVID-19 ONFH. Non-COVID-19 ONFH can be triggered by numerous risk factors, including fractures, steroid therapy, and alcohol abuse. Vascular disorders, which subsequently lead to the necrosis of bone cells, are one of the main mechanisms of the development of non-COVID-19 ONFH of various etiologies. In post-COVID-19 ONFH, mast cells appear to play a dominant role. These cells exhibit abnormal behavior when exposed to the coronavirus, undergoing excessive hyperplasia, activation, and degranulation. The substances secreted by mast cells subsequently trigger vascular dysfunction and thrombus formation while also promoting fibrosis development. This cascade of events not only initiates but also accelerates the progression of post-COVID-19 ONFH. The intensification of pathological processes is shown by multiple black arrows.

Thus, our data suggest that the key morphological biomarkers that trigger the main molecular and cellular mechanisms in the development of post-COVID-19 ONFH are clusters of mast cells, extensive areas of fibrosis, numerous arterial and venous thrombi, and giant cell granulomas. At the same time, the leading players are probably mast cells, which, when interacting with the COVID-19 virus, undergo excessive hyperplasia, activation and degranulation. The factors released by mast cells, in turn, increase inflammation, affect bone and cartilage metabolism, cause blood vessel dysfunction and thrombosis, and contribute to the development of fibrosis, thereby stimulating and facilitating the development of osteonecrosis of the femur (Figure 5). Our results demonstrate that the accumulation and activation of mast cells is a feature of the pathogenesis of post-COVID-19 osteonecrosis and can be considered as a diagnostic sign of this disease.

## Figures and Tables

**Table 1:** Characteristics of patients with diagnosed ONFH subjected to total arthroplasty.

	Non-COVID-19 (*n* = 47) M (SD)	Post-COVID-19 (*n* = 41) M (SD)	*p*-Value (One-Way ANOVA)
Age, years	53.7 (9.3)	51.2 (10.6)	0.907
Gender, %			
Male	63.2	58.6	0.522
Female	36.8	41.4	
BMI, kg/m^2^	29.7 (5.2)	28.6 (2.7)	0.986
Etiologies of ONFH, %			
tONFH	45.6	—	
sONFH	29.2		
aONFH	6.7		
iONFH	18.5		
ONFH duration, months	25.4 (7.2)	14.1 (9.2)	0.03
Interval from COVID-19 and onset of ONFH, months	—	11.3 (10.1)	
Steroid therapy for COVID-19, %			
Yes	—	16.8	
No		83.2	
Comorbidity, %	12.8	7.3	

Note: BMI, body mass index; ONFH, osteonecrosis of the femoral head; tONFH, traumatic ONFH; sONFH, steroid-induced ONFH; aONFH, alcohol-induced ONFH; iONFH, idiopathic ONFH; M (SD), mean (standard deviation).

## Data Availability

The data presented in this study are available upon request from the corresponding author.

## Abbreviations

The following abbreviations are used in this manuscript:

ONFH	Osteonecrosis of the Femoral Head
CPA3	Carboxypeptidase A3
ACE2	Angiotensin-Converting Enzyme 2
HRH1	Histamine Receptor H1
